# Altered skeletal muscle metabolic pathways, age, systemic inflammation, and low cardiorespiratory fitness associate with improvements in disease activity following high-intensity interval training in persons with rheumatoid arthritis

**DOI:** 10.1186/s13075-021-02570-3

**Published:** 2021-07-10

**Authors:** Brian J. Andonian, Andrew Johannemann, Monica J. Hubal, David M. Pober, Alec Koss, William E. Kraus, David B. Bartlett, Kim M. Huffman

**Affiliations:** 1 Division of Rheumatology and Immunology, Department of Medicine, Duke University School of Medicine, Durham, NC USA; 2 Duke Molecular Physiology Institute, Duke University School of Medicine, Durham, NC USA; 3 Department of Kinesiology, Indiana University-Purdue University Indianapolis School of Health & Human Sciences, Indianapolis, IN USA; 4 Phastar Inc., Cambridge, MA USA

**Keywords:** Rheumatoid arthritis, Skeletal muscle, Cardiorespiratory fitness, Metabolism, Gene expression, Disease activity, Inflammation, Exercise training

## Abstract

**Background:**

Exercise training, including high-intensity interval training (HIIT), improves rheumatoid arthritis (RA) inflammatory disease activity via unclear mechanisms. Because exercise requires skeletal muscle, skeletal muscle molecular pathways may contribute. The purpose of this study was to identify connections between skeletal muscle molecular pathways, RA disease activity, and RA disease activity improvements following HIIT.

**Methods:**

RA disease activity assessments and vastus lateralis skeletal muscle biopsies were performed in two separate cohorts of persons with established, seropositive, and/or erosive RA. Body composition and objective physical activity assessments were also performed in both the cross-sectional cohort and the longitudinal group before and after 10 weeks of HIIT. Baseline clinical assessments and muscle RNA gene expression were correlated with RA disease activity score in 28 joints (DAS-28) and DAS-28 improvements following HIIT. Skeletal muscle gene expression changes with HIIT were evaluated using analysis of covariance and biological pathway analysis.

**Results:**

RA inflammatory disease activity was associated with greater amounts of intramuscular adiposity and less vigorous aerobic exercise (both *p* < 0.05). HIIT-induced disease activity improvements were greatest in those with an older age, elevated erythrocyte sedimentation rate, low cardiorespiratory fitness, and a skeletal muscle molecular profile indicative of altered metabolic pathways (*p* < 0.05 for all). Specifically, disease activity improvements were linked to baseline expression of RA skeletal muscle genes with cellular functions to (1) increase amino acid catabolism and interconversion (GLDC, BCKDHB, AASS, PYCR, RPL15), (2) increase glycolytic lactate production (AGL, PDK2, LDHB, HIF1A), and (3) reduce oxidative metabolism via altered beta-oxidation (PXMP2, ACSS2), TCA cycle flux (OGDH, SUCLA2, MDH1B), and electron transport chain complex I function (NDUFV3). The muscle mitochondrial glycine cleavage system (GCS) was identified as critically involved in RA disease activity improvements given upregulation of multiple GCS genes at baseline, while GLDC was significantly downregulated following HIIT.

**Conclusion:**

In the absence of physical activity, RA inflammatory disease activity is associated with transcriptional remodeling of skeletal muscle metabolism. Following exercise training, the greatest improvements in disease activity occur in older, more inflamed, and less fit persons with RA. These exercise training-induced immunomodulatory changes may occur via reprogramming muscle bioenergetic and amino acid/protein homeostatic pathways.

**Trial registration:**

ClinicalTrials.gov, NCT02528344. Registered on 19 August 2015.

**Supplementary Information:**

The online version contains supplementary material available at 10.1186/s13075-021-02570-3.

## Introduction

Exercise training improves disease activity and systemic inflammation in rheumatoid arthritis (RA) [1–4]. In addition to traditional aerobic exercise programs, high-intensity interval training (HIIT)—where participants alternate bouts of near-maximal intensity aerobic exercise with bouts of lower intensity—significantly improves RA Disease Activity Score in 28 joints (DAS-28) and erythrocyte sedimentation rate (ESR) [5]. HIIT also significantly improves RA peripheral neutrophil chemotaxis and macrophage phagocytosis; findings suggest that exercise improves RA innate immune function and pathogen response [5]. In non-RA populations, chronic exercise training and lifelong physical activity improve regulatory T cell anti-inflammatory function, decrease CD4+ Th17 polarization, and ameliorate immunosenescence [6, 7]; however, the specific effects of exercise on improving RA chronic inflammation and adaptive immunity are unknown. Improved understanding of the mechanisms that connect exercise training with its immunomodulatory effects holds great promise to enhance future lifestyle and pharmacologic therapies for RA and other chronic inflammatory diseases.

One underexplored mediator of exercise-induced improvements in RA inflammation is skeletal muscle. Skeletal muscle is the predominant locomotive organ and is largely responsible for the beneficial health effects of exercise. Skeletal muscle, during acute exercise, communicates widely with other organ systems, including immune cells, via the secretion of proteins termed “myokines” [8]. Myokines have far-ranging benefits, including helping to regulate systemic energy metabolism [8–12]. For example, during exercise, interleukin (IL)-6 and IL-15 are secreted by muscle in a pulsatile fashion and, in contrast to sepsis or inflammation, independent from tumor necrosis factor (TNF)-α secretion. Muscle IL-6 secretion during exercise has direct anti-inflammatory effects by stimulating IL-10 production from CD4+ T cells and inhibiting systemic TNF-α secretion [13, 14]. While skeletal muscle contractile activity appears to improve immune function, chronic immune activation (i.e., RA) adversely impacts skeletal muscle.

In RA, chronic inflammation is strongly linked to impaired muscle function and “rheumatoid cachexia” or sarcopenic obesity (i.e., decreased muscle mass, increased fat mass, and intramuscular adiposity) [15]. Further, the RA skeletal muscle molecular profile is defined by altered inflammatory cytokines and impairments in satellite cell function and oxidative metabolism [15–18]. Of particular interest to this study, it is unclear how these RA muscle molecular pathways specifically (1) contribute to and are affected by inflammation and autoimmunity and (2) influence and predict immune system changes with exercise training. In a cross-sectional cohort of persons with established RA, we identified clinical and skeletal muscle molecular phenotypes associated with RA inflammatory disease activity. To better understand how exercise-trained skeletal muscle modulates inflammation, we then studied an independent RA cohort that completed a 10-week HIIT exercise program and identified baseline clinical and skeletal muscle molecular phenotypes associated with improvements in RA disease activity. We hypothesized that altered skeletal muscle immune and metabolic pathways are intricately linked to (1) RA inflammatory disease activity and (2) improvements in RA inflammation following exercise training.

## Methods

### Design and participants

For the cross-sectional RA cohort #1, persons with RA (*n* = 47) completed clinical assessments and underwent skeletal muscle biopsies in a cross-sectional design [16]. All RA participants met American College of Rheumatology (ACR) 1987 criteria [19]. Participants had a positive rheumatoid factor and/or anti-cyclic citrullinated peptide antibody or had imaging evidence of hand or foot erosions. They had no medication changes within 3 months of enrollment and were all using less than or equal to 5 mg of prednisone daily. RA participants were excluded with pregnancy, type 2 diabetes mellitus, or known cardiovascular disease.

For HIIT RA cohort #2, persons with RA (*n* = 12) completed clinical assessments and underwent skeletal muscle biopsies before and after a 10-week treadmill walking HIIT program [5, 17]. Similar to the cross-sectional RA cohort #1, all RA participants satisfied the ACR 1987 RA classification criteria [19], had no medication changes for 3 months prior to or during the intervention, and were taking less than or equal to prednisone 5 mg per day. RA medications used during the study were previously reported [17]. For the HIIT RA cohort #2, inclusion required participants to be sedentary, defined as exercising less than 2 days per week. Exclusions included diabetes mellitus, cardiovascular disease, and if they were unable to walk without assistance on a treadmill.

### Exercise intervention

In the HIIT RA cohort #2, persons with RA completed supervised treadmill walking exercise sessions three times per week for 10 weeks as previously described [5, 17]. Exercise prescriptions were based on baseline cardiopulmonary exercise tests. During each treadmill walking exercise session, participants completed (1) a 5-min warm-up, (2) 10 alternating intervals (60–90 s each) of high-intensity (80–90% heart rate reserve) and low-intensity (50–60% heart rate reserve) exercise, and (3) a 5-min cool-down.

### Clinical assessments

For RA cohorts #1 and #2, health history questionnaires were obtained and inflammatory disease activity was assessed by DAS-28 determined from patient-completed pain and overall health visual analog scales, physician assessed tender and swollen joint counts, and ESR [20]. For the cross-sectional RA cohort #1, physical activity was assessed via a wearable accelerometer for 7 days [16, 21]; body composition was assessed via abdominal and thigh CT scans [22]. For the HIIT RA cohort #2, cardiorespiratory fitness was assessed via graded treadmill exercise testing as maximal oxygen consumption during exercise (VO_2_ peak) [5, 16]; body composition was assessed via BodPod® (BodPod System; Life Measurement Corporation, Concord, CA, USA) [17].

### Skeletal muscle gene expression profiling

*Vastus lateralis* needle biopsies were obtained from participants after an overnight fast by standard Bergstrom technique and stored at −80°C in the cross-sectional RA cohort #1 and before and after 10 weeks of HIIT in RA cohort #2 at between 24 and 48 h after the last exercise training bout [17]. Illumina Human HT-12v3 and HT-12v4 Expression BeadChips were used for muscle quantitative RNA analyses for a random subset of cross-sectional RA cohort #1 (*n* = 20) and the HIIT RA cohort #2 (*n* = 12), respectively. For each frozen sample, 20–30 mg of the muscle was homogenized; biotinylated total RNA was prepared using the Illumina TotalPrep RNA amplification kit (Life Technologies, Grand Island, NY, USA) and hybridized to the Human HT-12 BeadChips (Illumina, San Diego, CA, USA); and RNA profiling performed as previously described [16].

### Statistical analyses

Differential Spearman correlations were performed using Partek Genomics Suite (Partek, Inc.; St. Louis, MO, USA) to assess associations of baseline muscle gene expression with (a) baseline DAS-28 in the cross-sectional RA cohort #1 and (b) HIIT-mediated change (i.e., improvement) in RA disease activity (ΔDAS-28) in the HIIT RA cohort #2. For the HIIT RA cohort #2, ΔDAS-28 was computed as pre-HIIT DAS-28 minus post-HIIT DAS-28 so that larger numbers and positive associations represent improved disease activity. To validate baseline DAS-28 correlations from the cross-sectional RA cohort #1, Spearman correlations were also performed between baseline muscle gene expression and baseline DAS-28 in the HIIT RA cohort #2. Genes with expression correlated with DAS-28 or ΔDAS28 (Spearman rho *p* < 0.05) were uploaded for Ingenuity Pathway Analyses (IPA, www.ingenuity.com); positive and negative coefficients represented positive and inverse associations, respectively, between gene expression and DAS-28 (cross-sectional RA cohort #1) or ΔDAS-28 (HIIT RA cohort #2). IPA-defined canonical pathways—created based on right-handed Tukey’s T tests to analyze overrepresentation of genes and pathways based on molecular pathways in the current literature—were identified as pathways with the greatest proportional representation (gene set/total pathway genes) (Fisher Exact Test; *p* < 0.05). Of importance to the interpretation of our analyses, we purposely chose a less stringent significance cutoff (i.e., *p* < 0.05 as opposed to false discovery rate) when creating initial gene sets to minimize random false positive and highly related results with downstream pathway analyses. Top differentially associated gene lists (*p* < 0.001) from both RA cohorts were analyzed for functional classification using the PANTHER Gene List Analysis tool and for overrepresentation against a *homo sapiens* gene reference database using the PANTHER Overrepresentation Test (http://pantherdb.org) with Reactome pathways (https://reactome.org) annotation [23–25]. Changes in gene expression fold differences after HIIT in RA cohort #2 were assessed via analysis of covariance (ANCOVA) modeling in Partek Genomics Suite.

## Results

### Clinical associations with RA disease activity

In the cross-sectional RA cohort #1, disease activity correlated with ESR (rho=0.80, *p* < 0.001), low thigh muscle density (i.e., greater intramuscular adipose tissue) [26] (rho=−0.32, *p* = 0.032), and fewer total daily minutes of high (rho=−0.36, *p* = 0.025) and very high intensity physical activity (rho=−0.37, *p* = 0.02) (Table 1).

**Table 1:** Rheumatoid arthritis clinical characteristics and disease activity relationships

Variable	Cross-sectional RA cohort #1 (***n*** = 47); Mean (SD)	Cross-sectional RA cohort #1: associations with DAS-28; Spearman’s rho	HIIT RA cohort #2 (***n*** = 12); Mean (SD)	HIIT RA cohort #2: associations with improvements in DAS-28 following HIIT; Spearman’s rho
DAS-28 (mean)				
Baseline	3.0 (1.4)		3.1 (1.5)	0.57
Post-HIIT			2.3 (1.5)	
ESR (mm/hr)	11.8 (12.0)	0.80*		
Baseline			10.5 (11.9)	0.64*
Post-HIIT			7.0 (8.8)	
CRP (mg/L)	6.5 (7.8)	0.47*		
Baseline			2.7 (3.5)	0.47
Post-HIIT			2.2 (3.1)	
Swollen joints (n)				
Baseline			4.3 (3.8)	0.74*
Post-HIIT			1.9 (1.7)	
Tender joints (n)				
Baseline			4.2 (7.0)	0.33
Post-HIIT			1.9 (2.9)	
Patient global health (mm)				
Baseline			31.5 (15.7)	−0.43
Post-HIIT			22.6 (19.2)	
HAQ-DI	0.67 (0.68)	0.67*		
Baseline			0.41 (0.34)	−0.13
Post-HIIT			0.35 (0.46)	
Age (years)	54.1 (12.5)	0.05	63.9 (7.2)	0.67*
BMI (kg/m^2^)	29.9 (6.4)	−0.01		
Baseline			27.4 (9.3)	0.32
Post-HIIT			27.7 (9.8)	
Gender				
Female	33 (70.2%)	0.07	11 (91.6)	0.10
Male	14 (29.8%)		1 (8.4%)	
Rheumatoid factor positive	42/47 (89.4%)	0.20	10/12 (83.3%)	−0.45
Anti-cyclic citrullinated antibody positive	21/22 (95.6%)	−0.21	5/8 (62.5%)	−0.29
Erosions on radiographs present	21/38 (55.2%)	0.24	9/12 (75.0%)	0.55
Disease duration (months)	138.9 (136.3)	−0.09	159.6 (86.7)	0.18
Body composition				
Abdominal total adipose area (cm^2^)	408.9 (202.6)	0.05		
Thigh total adipose area (cm^2^)	135.7 (66.8)	0.06		
Thigh muscle area (cm^2^)	115.8 (37.4)	−0.17		
Thigh muscle density (Hu)	53.4 (8.6)	−0.32*		
Body fat (%)				
Baseline			36.6 (11.6)	0.53
Post-HIIT			37.3 (11.2)	
Lean mass (kg)				
Baseline			44.9 (8.9)	0.11
Post-HIIT			44.7 (7.8)	
Sedentary activity (% total daily min)	91.0 (6.0)	0.23		
Total exercise (min/day)	9.7 (14.3)	−0.29		
Moderate intensity exercise (min/day)	7.5 (10.5)	−0.26		
High intensity exercise (min/day)	1.6 (3.8)	−0.36*		
Very high intensity exercise (min/day)	0.5 (2.9)	−0.37*		
Baseline cardiorespiratory fitness (VO_2_ peak; ml/kg/min)			24.9 (6.6) 27.1 (7.0)	−0.75*

In columns 2 and 4, data are presented as means (SD) for continuous variables and number (percentages) of participants for dichotomous variables. In columns 3 and 5, data are presented as Spearman’s rho

*RA* rheumatoid arthritis, *DAS-28* disease activity score in 28 joints, *HIIT* high-intensity interval training, *ESR* erythrocyte sedimentation rate, *CRP* c-reactive protein, *HAQ-DI* health assessment questionnaire-disability index, *BMI* body mass index

*p < 0.05 for Spearman correlations

### Skeletal muscle gene expression associations with RA disease activity

**Fig. 1:**
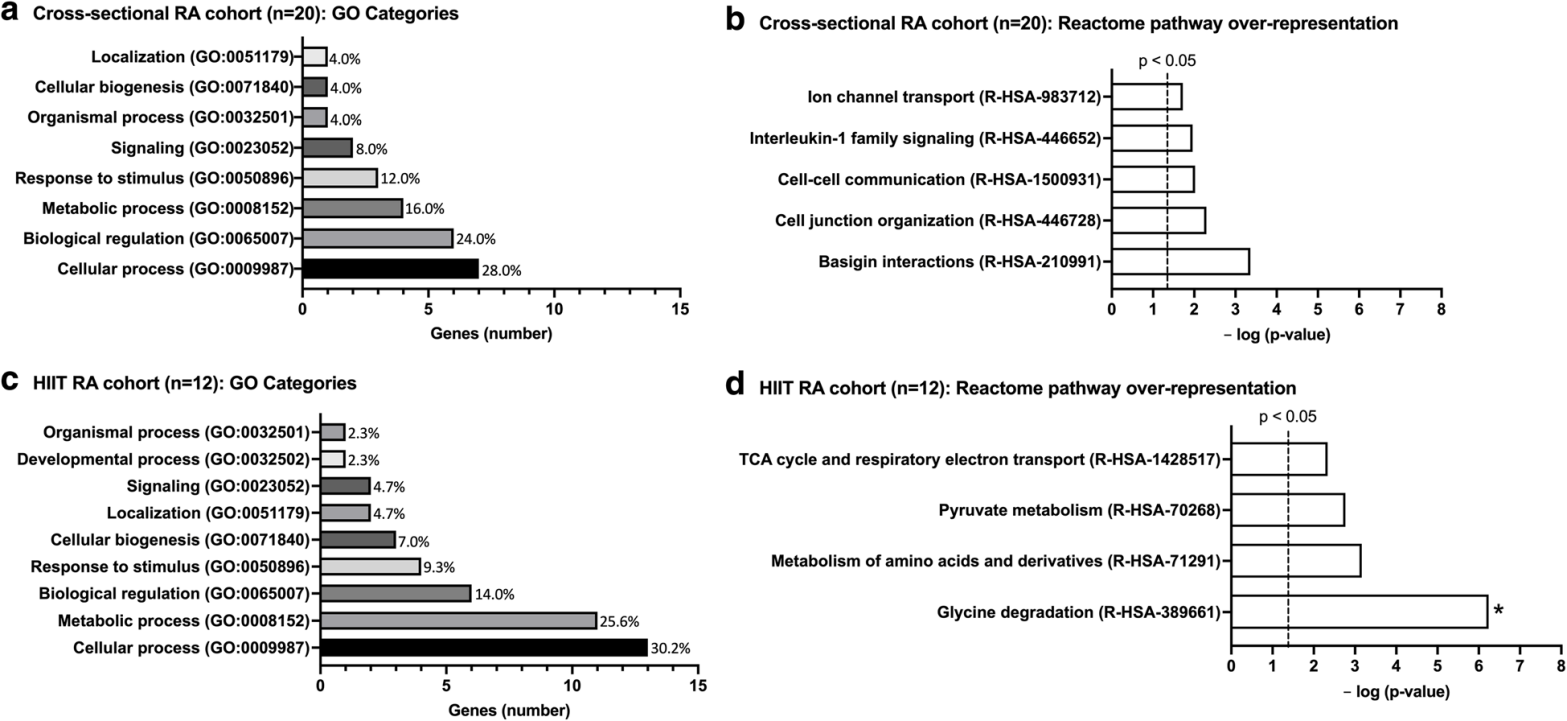
Top rheumatoid arthritis skeletal muscle gene associations with inflammatory disease activity. **a** Bar chart categorizing top rheumatoid arthritis (RA) skeletal muscle differentially expressed genes highly associated (Spearman’s rho *p* < 0.001) with RA disease activity score in 28 joints (DAS-28) by PANTHER gene ontology (GO) biological processes (bars represent total number genes per category; percent gene process hits/total listed next to bars). **b** Bar chart showing Reactome pathways overrepresented (>2 genes per pathway) within top RA skeletal muscle genes highly associated (*p* < 0.001) with disease activity. **c** Bar chart categorizing top baseline RA skeletal muscle differentially expressed genes highly associated (Spearman’s rho *p* < 0.001) with improvement in RA disease activity following high-intensity interval training (HIIT) by PANTHER GO biological processes (bars represent total number genes per category; percent gene process hits/total listed next to bars). **d** Bar chart showing Reactome pathways overrepresented (>2 genes per pathway) within top baseline RA skeletal muscle genes highly associated (*p* < 0.001) with improvement in disease activity following HIIT. *Represents pathway that reached significance at the calculated false discovery rate (*p* < 0.05)

Of 29,042 total mapped gene transcripts analyzed, expression of 25 genes was highly correlated (rho>0.68 or <−0.68; *p* < 0.001) with RA disease activity scores (DAS-28) in the cross-sectional RA cohort #1. The top highly correlated skeletal muscle gene was IL1RL2 (interleukin 1 receptor-like 2; rho=−0.84) (Supplementary Table 1). IL1RL2 expression in RA skeletal muscle was also significantly correlated with DAS-28 at baseline in the HIIT RA cohort #2 (Supplementary Table 1). The 25 highly correlated genes were categorized according to biological function (PANTHER gene ontology (GO)) (Fig. 1A) and pathways (Reactome) (Fig. 1B). Of the 25 highly associated genes, we identified Reactome pathways that were overrepresented. Without correction, 5 non-overlapping Reactome pathways were overrepresented (>2 genes/set) (*p* < 0.05): (1) Basigin interactions, (2) cell junction organization, (3) cell–cell communication, (4) interleukin-1 family signaling, and (5) ion channel transport. However, accounting for false discovery, no Reactome pathway was significantly overrepresented.

To further explore relationships between the skeletal muscle transcriptome and RA systemic inflammatory disease, we uploaded skeletal muscle genes differentially associated with disease activity to biological pathway software (IPA) (Spearman’s correlation *p* < 0.05). These data included a total of 1417 molecules (684 negatively correlated, 733 positively correlated). The top canonical pathway was “DNA Methylation and Transcriptional Repression Signaling” (Table 2 and Supplementary Table 2), represented by 7 genes (CHD4, MTA2, SIN3A, DNMT3A, DNMT3B, H4C14, MBD2) of 33 total genes in that pathway. Of the 10 top canonical pathways in the cross-sectional RA cohort #1, 5 included at least one significant skeletal muscle gene relationship that was validated in the HIIT RA cohort #2 (Supplementary Table 2).

**Table 2:** Cross-sectional rheumatoid arthritis cohort #1: skeletal muscle canonical pathways associated with disease activity

Ingenuity canonical pathways	– log (***p*** value)	Ratio (genes/total genes)	Positively associated genes	Negatively associated genes
DNA methylation and transcriptional repression signaling	2.18	7/33	CHD4,MTA2,SIN3A	DNMT3A,DNMT3B,H4C14,MBD2
tRNA splicing	2.06	8/43	PDE1C,PDE4B,PDE5A,PDE8A	MPPED2,PDE1A,PDE7A,SMPDL3B
Regulation of the epithelial mesenchymal transition in development pathway	1.96	12/82	AXIN1,GLI2,GLI3,JAG2,TCF4,WNT11,WNT8B, WNT9A	RBPJ,SNAI2,WNT3A,WNT5B
Sphingosine and sphingosine-1-phosphate metabolism	1.84	3/8	ACER3	ASAH1,SGPP1
TCA cycle II (eukaryotic)	1.65	5/24	IDH3B,IDH3G	DLD,MDH1B,SUCLA2
Branched-chain α-keto acid dehydrogenase complex^*#^	1.58	2/4	BCKDHB	DLD
Molybdenum cofactor biosynthesis	1.58	2/4		MOCS3,NFS1
D-myo-inositol (1,4,5)-trisphosphate biosynthesis	1.57	5/25	PI4KA,PIP4K2A,PIP5K1B	PI4K2A,PIP4K2B
Assembly of RNA polymerase I complex	1.44	3/11		POLR1B,TAF1A,TAF1C

Results of ingenuity canonical pathways analyses, with pathways reaching significance at *p* > 0.05 included. Ratio refers to the number of differentially expressed skeletal muscle genes significantly associated (Spearman correlations, *p* < 0.05) with rheumatoid arthritis (RA) (*n* = 20 participants) disease activity score in 28 joints (DAS-28) compared to the total number genes in that pathway. Subsequent significant pathways with at least 50% gene overlap from previous listed pathways are filtered from this table. Please see Supplementary Table 2 for full list of significant Canonical Pathways.

^*#^Pathway significantly associated in both the cross-sectional RA cohort #1 and changes with high-intensity interval training (HIIT) RA cohort #2 analyses

### Clinical associations with exercise training-related improvements in RA disease activity

In the HIIT RA cohort #2, HIIT-mediated improvements in DAS-28 correlated with older age (rho=0.67, *p* = 0.022), higher baseline ESR (rho=0.64, *p* = 0.035), and lower baseline cardiorespiratory fitness/VO_2_ peak (rho=−0.75, *p* = 0.001) (Table 1).

### Baseline skeletal muscle gene expression associations with exercise training-related improvements in RA disease activity

Muscle expression for 43 genes of 27,911 total mapped gene transcripts analyzed in the HIIT RA cohort #2 was highly correlated with improvements in disease activity (rho>0.80 or <−0.80; *p* < 0.001). The strongest correlation was for KIF25 (kinesin family member 25; rho=0.915) (Supplementary Table 3). Within the 43 genes, all 4 non-overlapping, overrepresented Reactome pathways (without correction; *p* < 0.05) involved cellular substrate energy metabolism: (1) glycine degradation, (2) metabolism of amino acids and derivatives, (3) pyruvate metabolism, and (4) tricarboxylic acid (TCA) cycle and respiratory electron transport. The glycine degradation pathway (R-HSA-389661) was the only pathway significantly overrepresented at the false discovery rate (Fig. 1D). Of genes where baseline expression was associated with HIIT-mediated improvement in disease activity, KIF25 (kinesin family member 25) and MNT (MAX network transcriptional repressor) were significantly upregulated and GLDC (glycine decarboxylase) was significantly downregulated following HIIT (*p* < 0.05) (Supplementary Table 3).

We used IPA to better understand skeletal muscle pathways that may contribute to exercise-induced immune regulation. Using a *p* value cut off of 0.05 for Spearman’s correlations, 1527 molecules (1049 negatively correlated, 478 positively correlated) were uploaded. IPA identified 39 canonical pathways (*p* < 0.05). The top canonical pathway was “Proline Biosynthesis II” (Table 3 and Supplementary Table 4), represented by 4 genes (OAT, PYCR1, PYCR2, PYCR3) of 5 total genes in that pathway. The top canonical pathway not directly related to energy substrate metabolism was “nNOS Signaling in Skeletal Muscle Cells” (Table 3), which included 9 (CACNA1A, CACNA1C, CACNA1I, CACNA2D4, CACNB4, CACNG8, CHRNA1, RYR1, CACNB1) of 40 pathway genes. The only canonical pathway represented in both analyses for RA cohorts #1 and #2 was “Branched-chain α-keto acid Dehydrogenase Complex” (Tables 2 and 3).

**Table 3:** High-intensity interval training rheumatoid arthritis cohort #2: skeletal muscle canonical pathways associated with improvements in disease activity

Ingenuity canonical pathways	– log (***p*** value)	Ratio (genes/total genes)	Positively associated genes	Negatively associated genes
Proline biosynthesis II	3.84	4/5	OAT,PYCR1,PYCR2,PYCR3	
Glycine cleavage complex	3.38	4/6	AMT,GCSH,GLDC,TBXT	
Purine biosynthesis II	3.14	5/11	ADSS1,IMPDH1,PAICS	ADSS2,IMPDH2
Proline biosynthesis I	2.8	3/4	PYCR1,PYCR2,PYCR3	
nNOS signaling in skeletal muscle cells	2.63	9/40	CACNA1A,CACNA1C,CACNA1I,CACNA2D4, CACNB4,CACNG8,CHRNA1,RYR1	CACNB1
Sertoli cell-sertoli cell junction signaling	2.36	24/181	CLDN14,CLDN18,CLDN19,ITGA3,MAP2K3,MYO7A,PRKAG2,PRKG2,SYMPK,TJAP1,TUBB8	ACTC1,LK,MAP3K6,MAPK9,MRAS,NECTIN1, RASD1,RRAS,SORBS1,SPTBN1,TUBA1A,TUBB2A, TUBB2B
L-DOPA degradation	2.25	2/2	COMT,LRTOMT	
VDR/RXR activation	1.94	12/77	HOXA10,HSD17B2,IGFBP3,IGFBP5,KLK6,NCOR2, RXRB,SEMA3B,SULT2A1,WT1	CASR,TRPV6
Ethanol degradation II	1.88	6/27	ADH1C,AKR1A1,ALDH3A1,ALDH9A1	ACSS2,ALDH7A1
Estrogen receptor signaling	1.84	35/319	AGT,ATP5F1A,ATP5PB,BCL2,CACNA1A,CACNA1C,CTBP1,EGFR,ESR1,GNAO1,HIF1A,MED10,MED14, MED16,MED4, MED6, NCOR2,PLCB1,PRKAG2, TFAM,TYK2,VEGFC	CTBP2,EGF,FOXO6,MMP19,MMP20,MMP21,MRAS,MYL1,MYL6,PLCB2, POLR2B,RASD1,RRAS
HIF1α signaling	1.84	24/200	APEX1,ARAF,BMP6,CAMK1D,HIF1A,HSPA4, HSPA6,LDHB,MAP2K3,MKNK2,MMP19,MMP20, SLC2A14,SLC2A3,VEGFC	CCNG2,EGF,MDM2,MMP21,MRAS,PPP3R1,RAN, RASD1,RRAS
Methylglyoxal degradation III	1.77	4/14	AKR1A1,DHRS11	AKR1C1/AKR1C2,AKR1C3
Estrogen-dependent breast cancer signaling	1.72	11/73	DHRS11,EGFR,ESR1,HSD17B11,HSD17B12, HSD17B2	AKR1C4,MRAS,RASD1,RRAS,TERT
Vitamin-C transport	1.6	5/23	NXN,SLC2A3,TXNRD3	AKR1C4,LRRC8A
2-KG dehydrogenase complex	1.52	2/4	DLST	OGDH
Branched-chain α-keto acid dehydrogenase complex^*#^	1.52	2/4	BCKDHB,DBT	
Neuroprotective role of THOP1 in Alzheimer’s disease	1.49	14/109	AGT,APP,GNRH2,GZMA,KLK12,KLK6,PRKAG2, PRSS3,SERPINA3	FAP,HLA-B,PRSS23,ST14,YWHAE
Glycogen degradation II	1.36	3/11	AGL,PGM5	TYMP

Results of ingenuity canonical pathways analyses, with pathways reaching significance at *p* > 0.05 included. Ratio refers to the number of differentially expressed skeletal muscle genes significantly associated (Spearman correlations, *p* < 0.05) with improvements in rheumatoid arthritis (RA) (RA) (*n* = 12 participants) disease activity score in 28 joints (DAS-28) following high-intensity interval training (HIIT) compared to the total number genes in that pathway. Subsequent significant pathways with at least 50% gene overlap from previous listed pathways are filtered from this table. Please see Supplementary Table 4 for full list of significant canonical pathways

^*#^Pathway significantly associated in both the cross-sectional RA cohort #1 and changes with high-intensity interval training (HIIT) RA cohort #2 analyses

## Discussion

**Fig. 2:**
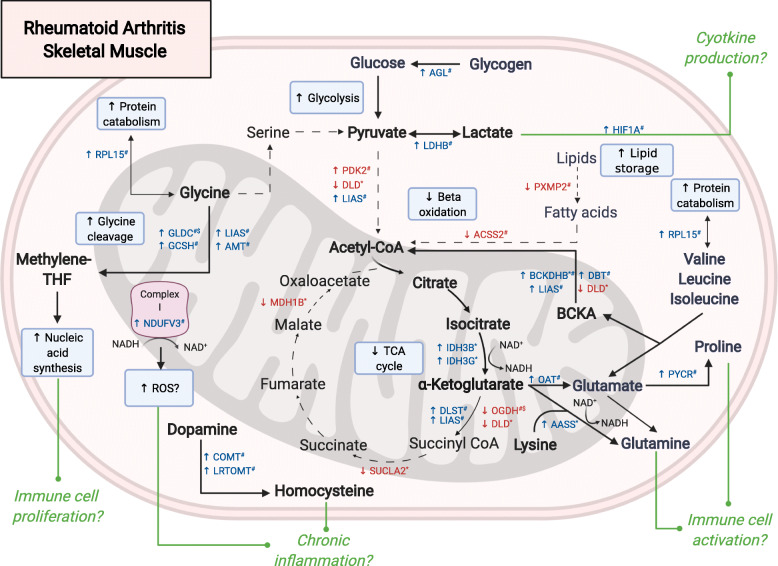
Is altered skeletal muscle metabolism linked to fueling chronic inflammation? Figure summarizes skeletal muscle cellular metabolic pathways that may contribute to rheumatoid arthritis (RA) chronic inflammation based on differentially expressed RA muscle genes that are highly associated with disease activity score in 28 joints (DAS-28) (*) or improvements in DAS-28 following high-intensity interval training (HIIT) (**#**). Genes included in the pathways diagram are either highly associated (Spearman’s rho *p* < 0.001) or part of significantly associated ingenuity canonical pathways (*p* < 0.05). Genes whose expression significantly (*p* < 0.05) changes following HIIT are listed ($). Based on direction of positive (↑) or negative (↓) associations, genes whose function is to promote/upregulate (blue) or inhibit/downregulate (red) a specific pathway are highlighted. Bolded arrows represent proposed upregulated pathways. Dashed arrows represent proposed downregulated pathways. Green end-dotted arrows represent hypothesized pathways in which altered skeletal muscle metabolism may contribute to chronic immune-activation and inflammation in RA

Via analysis of two cohorts of established RA (one cross-sectional and one prior to and after exercise training), we identified a phenotype for an exercise training-induced anti-inflammatory response as being older, more inflamed, less aerobically fit, and with multiple alterations in skeletal muscle metabolic pathways. Based on our transcriptomic analyses of top highly associated genes and IPA canonical pathways, we have identified the apparent skeletal muscle cellular metabolic pathways that (1) are connected to RA inflammation and (2) are associated with exercise training modulation of systemic immune responses (Fig. 2). These skeletal muscle pathways are highlighted by major gene expression alterations in amino acid catabolism and the regulation of glycolysis and TCA cycle flux. Taken together, rewiring of protein homeostasis and oxidative metabolism in sedentary RA skeletal muscle may be connected to the perpetuation of systemic inflammation. This association of the RA muscle transcriptional profile with HIIT-mediated improvements in RA disease activity suggests that exercise modulation of inflammation occurs in concert with the reprogramming of skeletal muscle metabolism.

### Amino acid homeostasis

In RA, amino acids are critical to sustain chronic inflammation. In RA synovium, chronic inflammation upregulates amino acid transporters, fueling maladaptive synovial proliferation and bone remodeling pathways [27]. In the circulation, activated RA immune cells have high energy demands that are met with glutamine, the majority of which is produced by skeletal muscle [28–30]. Further, inhibition of glutaminolysis is a promising therapeutic target for the management of Th17-driven autoimmune diseases [31, 32]. Our data demonstrate that muscle catabolism and interconversion of multiple amino acids—including glycine, lysine, glutamate/glutamine, and the branched-chain amino acids leucine, isoleucine, and valine—are linked to both RA inflammation and exercise training-induced reduction of inflammation (Fig. 2).

Chronic immune activity and exercise training-related modulation appear strongly related to the RA muscle mitochondrial glycine cleavage pathway. Baseline upregulation of multiple key glycine cleavage system genes (GLDC, GCSH, LIAS, AMT) associated with HIIT-related improvements in disease activity, while GLDC (glycine dehydrogenase) was significantly downregulated following HIIT. Glycine is a proteogenic amino acid and critical for multiple metabolic pathways in humans [33]. Catabolism of glycine via the glycine cleavage pathway to *N*^5^,*N*^10^-methylenetetrahydrofolate is utilized for the biosynthesis of purine nucleotides [34, 35], key regulators of T lymphocyte proliferation and survival [36, 37]. Thus, increased activity of the glycine cleavage system may be one way that impaired RA skeletal muscle metabolism contributes to and is impacted by chronic RA immune dysfunction.

Altered skeletal muscle metabolism of other amino acids also link to RA inflammation. Skeletal muscle branched-chain amino acid (BCAA) catabolism—via increased mitochondrial branched-chain α-ketoacid (BCKA) dehydrogenase (BCKDH) complex activity—was associated with both baseline RA disease activity and with exercise-mediated disease activity improvements. We theorize that increased RA muscle BCKDH activity might lead to glutamate/glutamine formation via multiple pathways. First, BCAA are metabolized to both BCKA and glutamate directly. Second, BCKA are converted to acetyl-CoA; subsequent α-ketoglutarate flux from the TCA cycle leads to further glutamate production via ornithine aminotransferase/OAT [25, 34]. Glutamate is then readily converted to glutamine with glutamine production augmented by lysine degradation via increased muscle AACC activity. Glutamine released into circulation can then sustain activation of RA immune cells [30, 34, 38] (Fig. 2).

Interestingly, opposing protein homeostatic pathways involved in both protein synthesis and muscle atrophy associated with subsequent improvement in RA inflammatory disease activity following HIIT. These pathways were highlighted by greater gene expression of RPL15 (a ribosomal protein critical for muscle protein synthesis) [34] and the nNOS calcium signaling canonical pathway important for driving muscle atrophy [39]. Taken together, these altered pathways suggest that RA skeletal muscle tissue is in a heightened state of protein turn-over; further, exercise training may work in part to regulate these protein catabolic and anabolic programs. Our findings of upregulated RA muscle protein turnover are in agreement with the prominent clinical phenotype of muscle loss due to “rheumatoid cachexia” or sarcopenic obesity [15]. Therefore, the increased ribosomal activity in RA muscle may represent a failed compensatory response to maintain muscle mass in the face of constant protein breakdown and atrophy signaling triggered by chronic inflammation. One unanswered question is whether this state of dysregulated RA muscle amino acid/protein homeostasis is merely a byproduct of inflammation or if muscle protein breakdown truly contributes to direct fueling of chronic immune activity [30].

### Glycolysis regulation

In addition to amino acids, dysregulation of RA skeletal muscle glycolysis and lactate production appears to support chronic immune signaling. We previously showed that, as compared to healthy matched controls, RA skeletal muscle is characterized by increased glycolysis as evidenced by transcriptional downregulation of oxidative metabolism components and accumulation of pyruvate [16]. Here, in a separate RA cohort, additional pro-glycolytic programs were linked to exercise-mediated improvements in RA disease activity. These transcriptomic features include increased pre-training breakdown of glycogen to glucose, decreased conversion of pyruvate to acetyl CoA, and increased interconversion between pyruvate and lactate [34] (Fig. 2). It is unclear from our data alone if an increased reliance on muscle glycolysis and subsequent lactate production contributes to RA immune cytokine signaling. Of note, the release of lactate from tumor and inflamed tissue—including RA synovium—stimulates pro-inflammatory IL-17 secretion [40–42]. Further, our data show that increased RA muscle hypoxia-inducible factor 1 alpha (HIF1A) expression is also associated with exercise-related improvements in inflammation; increased lactate can trigger increased HIF1A [43], where HIF1A can then stimulate immune cell IL-1β and IL-17 production [44].

### TCA cycle flux and oxidative metabolism

Increased RA muscle glycolysis appears to be connected to remodeling of oxidative metabolic pathways. Oxidative phosphorylation requires reducing agents (i.e., NADH and FADH_2_), typically generated through acetyl CoA and TCA cycle metabolism. Our findings suggest that RA muscle amino acid sources contribute more and glucose and fatty acids less than expected to acetyl CoA generation (Fig. 2). Remodeling of RA muscle oxidative metabolism was also evidenced by the downregulation of multiple TCA cycle enzymes (i.e., OGDH, SUCLA2, MDH1B). This apparent reduction in beta-oxidation is congruent with findings of increased lipid storage (i.e., intramuscular adiposity) in RA muscle. Intramuscular adiposity is linked to disability and an early aging phenotype in RA [45, 46]. Taken together, this suggests that exercise training is potentially important for modulating both RA fat metabolism and functional impairments.

### Immune pathways

In addition to metabolic alterations, we hypothesized that upregulated RA skeletal muscle immune/inflammatory cytokine pathways would be associated with disease activity and improvements in disease activity following exercise training; however, our data did not consistently support this theory. In the cross-sectional RA cohort #1, IL1RL2 and TAB1 were the only skeletal muscle genes specifically involved in immune function or inflammation to correlate highly with RA disease activity (Supplementary Table 1). Skeletal muscle IL-15 production was the top canonical pathway associated with disease activity (12/128 genes; *p* = 0.12; data not shown). Interestingly, muscle IL-15 has important roles in regulating fat mass and systemic metabolism, as well as modulating lymphocyte development and acute inflammation [11]. In the HIIT RA cohort #2, only muscle expression of immune-related genes FCRL6, TNFRSF19, CMTM4, and NKG7 were highly correlated with improvements in RA disease activity following HIIT. In established RA, these data suggest that systemic inflammation impacts skeletal muscle metabolism to a greater extent than the direct effects of localized inflammation within skeletal muscle tissue.

### Limitations

Though we have identified multiple skeletal muscle pathways that are connected to RA inflammation and can potentially be modulated by exercise training, our findings should be reviewed in the context of a few key limitations. Primarily, the bulk of our analyses relied on correlations based on stored human tissue samples; and thus, true causative pathways or intervenable targets could not be interrogated. Further, in the HIIT RA cohort #2, we analyzed correlations between baseline/pre-HIIT factors—as opposed to changes in these factors—with changes in RA disease activity following exercise training. While this decision to focus on baseline factors may limit our understanding of co-occurring exercise training-induced muscle and immune modifications, our analyses better show how immune overactivity influences RA muscle at baseline and the potential for exercise training to rectify those interactions. The longitudinal HIIT RA cohort analyses are also limited by the small sample size of 12 RA participants and the lack of a non-RA control group completing the HIIT program; thus, it is unclear if the findings of the study are unique to RA or not. Finally, our HIIT cohort did not include a control RA group (i.e., not undergoing exercise training) which somewhat limits the interpretation of exercise training-specific effects on RA muscle and immune function. Thus, further detailed study of cross-talk between RA skeletal muscle and immune cells is necessary to dissect the intricacies of these pathways so they can eventually be exploited to improve patient care.

## Conclusion

High-intensity interval training yielded the greatest improvements in inflammatory disease activity for persons with RA who were older, less aerobically fit, and with greater systemic inflammation. We identified alterations in multiple RA skeletal muscle metabolic pathways that associate strongly with disease activity as well as exercise training-related improvements in disease activity. Our data suggest that transcriptional remodeling of RA muscle amino acid, glucose, and fat metabolism is intricately linked to chronic inflammation. Exercise training may reprogram these muscle metabolic pathways to help maintain muscle-immune bioenergetic balance and modulate systemic immune responses. Ultimately, our findings provide further evidence for connections between impaired skeletal muscle and immune function in chronic inflammatory diseases such as RA and the ability of exercise training to rectify these underappreciated interorgan maladaptations.

## Supplementary Information

**Additional file 1: Supplementary Table 1.** Cross-sectional rheumatoid arthritis cohort #1 top skeletal muscle genes. **Supplementary Table 2.** Cross-sectional rheumatoid arthritis cohort #1: Skeletal muscle canonical pathways associated with disease activity. **Supplementary Table 3.** High-intensity interval training rheumatoid arthritis cohort #2 top skeletal muscle genes associated with improvements in disease activity. **Supplementary Table 4.** High-intensity interval training rheumatoid arthritis cohort #2: Skeletal muscle canonical pathways associated with improvements in disease activity.

## Data Availability

The raw data supporting the conclusions of this article will be made available by the authors upon request without undue reservation. Illumina array data will be deposited to the National Center for Biotechnology Information (NCBI) Gene Expression Omnibus (GEO).
