# Supplementary material for: Altered skeletal muscle metabolic pathways, age, systemic inflammation, and low cardiorespiratory fitness associate with improvements in disease activity following high-intensity interval training in persons with rheumatoid arthritis
Source: Arthritis Res Ther. 2021 Jul 10;23:187. doi: 10.1186/s13075-021-02570-3 (PMC8272378; doi:10.1186/s13075-021-02570-3)
Supplement: Supplementary file 1 — Additional file 1: Supplementary Table 1. Cross-sectional rheumatoid arthritis cohort #1 top skeletal muscle genes. Supplementary Table 2. Cross-sectional rheumatoid arthritis cohort #1: Skeletal muscle canonical pathways associated with disease activity. Supplementary Table 3. High-intensity interval training rheumatoid arthritis cohort #2 top skeletal muscle genes associated with improvements in disease activity. Supplementary Table 4. High-intensity interval training rheumatoid arthritis cohort #2: Skeletal muscle canonical pathways associated with improvements in disease activity. [file 13075_2021_2570_MOESM1_ESM.docx]

Supplementary Table 1. Cross-sectional rheumatoid arthritis cohort #1 top skeletal muscle genes

| **Symbol** | **Entrez gene name** | **Molecular process** | **Biological process** | **Cellular component** | **Association with RA DAS-28**  **(Spearman’s Rho)** |
| --- | --- | --- | --- | --- | --- |
| IL1RL2^†^ | interleukin 1 receptor like 2 | hydrolase, receptor | immunity, inflammatory response, innate immunity | plasma membrane | -0.841 |
| OR6C74 | olfactory receptor family 6 subfamily C member 74 | G-protein coupled receptor, receptor, transducer | olfaction, sensory transduction | plasma membrane | 0.803 |
| GREB1 | growth regulating estrogen receptor binding 1 | developmental protein | n/a | n/a | 0.798 |
| WFDC2 | WAP four-disulfide core domain 2 | aspartic protease inhibitor, protease inhibitor, serine protease inhibitor, thiol protease inhibitor | n/a | extracellular | 0.785 |
| TAB1 | TGF-beta activated kinase 1 (MAP3K7) binding protein 1 | n/a | n/a | cytosol, endosome, nucleus | 0.764 |
| DDX59 | DEAD-box helicase 59 | helicase, hydrolase, RNA-binding | n/a | nucleus | -0.759 |
| OR5AS1 | olfactory receptor family 5 subfamily AS member 1 | G-protein coupled receptor, receptor, transducer | olfaction, sensory transduction | plasma membrane | 0.755 |
| CZIB | CXXC motif containing zinc binding protein | n/a | n/a | n/a | 0.750 |
| CCDC80 | coiled-coil domain containing 80 | n/a | n/a | extracellular | 0.740 |
| TUSC3 | tumor suppressor candidate 3 | n/a | transport | endoplasmic reticulum, mitochondrion, plasma membrane | -0.725 |
| PPM1E | protein phosphatase, Mg2+/Mn2+ dependent 1E | hydrolase, protein phosphatase | n/a | mitochondrion, nucleus | 0.720 |
| MPZL1 | myelin protein zero like 1 | n/a | n/a | plasma membrane | -0.720 |
| ADAM32 | ADAM metallopeptidase domain 32 | n/a | n/a | plasma membrane | 0.719 |
| MPP5 | membrane palmitoylated protein 5 | n/a | n/a | extracellular, golgi apparatus, plasma membrane | 0.714 |
| CDY1 | chromodomain Y-linked 1 | acyltransferase, transferase | n/a | nucleus | 0.713 |
| ADORA3 | adenosine A3 receptor | G-protein coupled receptor, receptor, transducer | n/a | plasma membrane | 0.713 |
| ATP1B1 | ATPase Na+/K+ transporting subunit beta 1 | n/a | cell adhesion, ion transport, potassium transport, sodium transport, sodium/potassium transport, transport | extracellular, plasma membrane | -0.712 |
| SMYD4 | SET and MYND domain containing 4 | methyltransferase, transferase | n/a | n/a | 0.711 |
| DDX11 | DEAD/H-box helicase 11 | activator, developmental protein, DNA-binding, helicase, hydrolase, RNA-binding | DNA damage, DNA repair, DNA replication, host-virus interaction, transcription, transcription regulation | cytoskeleton, nucleus | 0.705 |
| CNGA3 | cyclic nucleotide gated channel subunit alpha 3 | ion channel, ligand-gated ion channel | ion channel, sensory transduction, transport, vision | plasma membrane | 0.702 |
| CSNK1G2-AS1 | CSNK1G2 antisense RNA 1 | n/a | n/a | n/a | -0.698 |
| MLKL | mixed lineage kinase domain like pseudokinase | n/a | necrosis | cytosol, nucleus, plasma membrane | 0.695 |
| EFCAB13 | EF-hand calcium binding domain 13 | n/a | n/a | n/a | -0.692 |
| RLBP1 | retinaldehyde binding protein 1 | n/a | sensory transduction, transport, vision | cytoskeleton, cytosol, nucleus | 0.686 |
| ITGA6 | integrin subunit alpha 6 | integrin, receptor | cell adhesion | extracellular, plasma membrane | -0.681 |

Results of top (p<0.001) Spearman correlation analyses between differentially expressed skeletal muscle genes with rheumatoid arthritis (RA) (n=20 participants) disease activity score in 28 joints (DAS-28). Gene molecular process, biological process, and cellular component classifications were identified using Uniprot (www.uniprot.org) keywords. n/a not available

^†^Skeletal muscle gene relationship with RA DAS-28 validated with corresponding correlation in baseline data from high-intensity interval training RA cohort #2 (n=12) with p<0.05.

Supplementary Table 2. Cross-sectional rheumatoid arthritis cohort #1: Skeletal muscle canonical pathways associated with disease activity

| **Ingenuity**  **Canonical**  **Pathways** | **– log**  **(p-value)** | **Ratio (genes/total genes)** | **Positively associated genes** | **Negatively associated genes** |
| --- | --- | --- | --- | --- |
| DNA Methylation and Transcriptional Repression Signaling | 2.18 | 7/33 | CHD4,MTA2,SIN3A | DNMT3A,DNMT3B,H4C14^†^,MBD2 |
| tRNA Splicing | 2.06 | 8/43 | PDE1C,PDE4B^†^,PDE5A,PDE8A | MPPED2,PDE1A,PDE7A,SMPDL3B |
| Regulation of The Epithelial Mesenchymal Transition in Development Pathway | 1.96 | 12/82 | AXIN1,GLI2,GLI3,JAG2,TCF4,WNT11,WNT8B, WNT9A | RBPJ,SNAI2,WNT3A,WNT5B |
| Sphingosine and Sphingosine-1-phosphate Metabolism | 1.84 | 3/8 | ACER3 | ASAH1,SGPP1 |
| Basal Cell Carcinoma Signaling | 1.65 | 10/70 | AXIN1,BMP1^†^,GLI2,GLI3,TCF4,WNT11,WNT8B, WNT9A | WNT3A,WNT5B |
| TCA Cycle II (Eukaryotic) | 1.65 | 5/24 | IDH3B,IDH3G | DLD,MDH1B,SUCLA2 |
| Branched-chain α-keto acid Dehydrogenase Complex**^*#^** | 1.58 | 2/4 | BCKDHB | DLD |
| Molybdenum Cofactor Biosynthesis | 1.58 | 2/4 |  | MOCS3^†^,NFS1 |
| D-myo-inositol (1,4,5)-Trisphosphate Biosynthesis | 1.57 | 5/25 | PI4KA,PIP4K2A,PIP5K1B | PI4K2A,PIP4K2B |
| Assembly of RNA Polymerase I Complex | 1.44 | 3/11 |  | POLR1B,TAF1A^†^,TAF1C |

Results of Ingenuity Canonical Pathways analyses, with pathways reaching significance at p>0.05 included. Ratio refers to the number of differentially expressed skeletal muscle genes significantly associated (Spearman correlations, p<0.05) with rheumatoid arthritis (RA) (n=20 participants) disease activity score in 28 joints (DAS-28) compared to the total number genes in that pathway.

**^*#^**Pathway significantly associated in both the cross-sectional RA cohort #1 and changes with high-intensity interval training (HIIT) RA cohort #2 analyses

^†^Skeletal muscle gene relationship with RA DAS-28 validated with corresponding correlation in baseline data from HIIT RA cohort #2 (n=12) with p<0.05.

Supplementary Table 3. High-intensity interval training rheumatoid arthritis cohort #2 top skeletal muscle genes associated with improvements in disease activity

| **Symbol** | **Entrez gene name** | **Molecular process** | **Biological process** | **Cellular component** | **Association with RA**  **ΔDAS-28**  **(Spearman’s Rho)** | **Δ Fold Change**  **(post-HIIT – pre-HIIT)** |
| --- | --- | --- | --- | --- | --- | --- |
| KIF25 | kinesin family member 25 | motor protein | n/a | cytoskeleton | 0.915 | **1.053*** |
| CCDC144A | coiled-coil domain containing 144A | n/a | n/a | n/a | 0.909 | -1.013 |
| AGBL1 | ATP/GTP binding protein like 1 | n/a | n/a | cytosol | 0.896 | -1.012 |
| RAB11FIP5 | RAB11 family interacting protein 5 | n/a | protein transport, transport | cytoskeleton, endosome, golgi apparatus, mitochondrion | 0.895 | -1.035 |
| FCRL6 | Fc receptor like 6 | receptor | n/a | plasma membrane | 0.888 | -1.010 |
| LIAS | lipoic acid synthetase | transferase | n/a | mitochondrion | 0.880 | 1.043 |
| GSE1 | Gse1 coiled-coil protein | n/a | n/a | n/a | 0.875 | -1.042 |
| TBXT | T-box transcription factor T | activator, developmental protein, DNA-binding | transcription, transcription regulation | nucleus | 0.870 | 1.006 |
| POGLUT3 | protein O-glucosyltransferase 3 | glycosyltransferase, transferase | n/a | endoplasmic reticulum | -0.867 | -1.017 |
| TOGARAM2 | TOG array regulator of axonemal microtubules 2 | n/a | n/a | cytoskeleton | 0.863 | 1.077 |
| TMCC2 | transmembrane and coiled-coil domain family 2 | n/a | n/a | endoplasmic reticulum | 0.858 | 1.0001 |
| ZNF91 | zinc finger protein 91 | DNA-binding, repressor | transcription, transcription regulation | nucleus | 0.849 | 1.047 |
| NDUFV3 | NADH:ubiquinone oxidoreductase subunit V3 | n/a | electron transport, respiratory chain, transport | mitochondrion | 0.847 | 1.040 |
| GLDC | glycine decarboxylase | oxidoreductase | n/a | mitochondrion | 0.845 | **-1.043*** |
| ZSWIM7 | zinc finger SWIM-type containing 7 | n/a | DNA damage, DNA recombination, DNA repair | nucleus | -0.840 | 1.020 |
| AGL | amylo-alpha-1, 6-glucosidase, 4-alpha-glucanotransferase | glycosidase, glycosyltransferase, hydrolase, multifunctional enzyme, transferase | glycogen biosynthesis | cytosol, endoplasmic reticulum, extracellular region, nucleus | 0.838 | 1.077 |
| LGALSL | galectin like | n/a | n/a | n/a | 0.838 | -1.083 |
| TNFRSF19 | TNF receptor superfamily member 19 | receptor | n/a | plasma membrane | 0.838 | -1.009 |
| ELP2 | elongator acetyltransferase complex subunit 2 | n/a | transcription, transcription regulation, tRNA processing | cytosol, nucleus | 0.837 | -1.021 |
| MNT | MAX network transcriptional repressor | DNA-binding, repressor | transcription, transcription regulation | nucleus | 0.837 | **1.064*** |
| PARD6G | par-6 family cell polarity regulator gamma | n/a | cell cycle, cell division | cytosol, nucleus, plasma membrane | 0.836 | -1.013 |
| CAND2 | cullin associated and neddylation dissociated 2 (putative) | n/a | transcription, Ubl conjugation pathway | cytosol, nucleus | 0.836 | -1.063 |
| ANAPC1P2 | ANAPC1 pseudogene 2 | n/a | n/a | cytosol, nucleus | -0.834 | 1.029 |
| TRPC4AP | transient receptor potential cation channel subfamily C member 4 associated protein | n/a | Ubl conjugation pathway | plasma membrane | 0.831 | 1.013 |
| BCKDHB | branched chain keto acid dehydrogenase E1 subunit beta | oxidoreductase | n/a | mitochondrion | 0.830 | 1.019 |
| CMTM4 | CKLF like MARVEL transmembrane domain containing 4 | n/a | n/a | n/a | 0.829 | -1.038 |
| HAX1 | HCLS1 associated protein X-1 | n/a | n/a | cytoskeleton, endoplasmic reticulum, mitochondrion, nucleus, plasma membrane | 0.829 | -1.018 |
| RBBP4 | RB binding protein 4, chromatin remodeling factor | chromatin regulator, Repressor | cell cycle, DNA replication, transcription, transcription regulation | cytosol, nucleus | 0.827 | -1.070 |
| ZNF263 | zinc finger protein 263 | DNA-binding, repressor | transcription, transcription regulation | nucleus | -0.826 | 1.010 |
| ARRDC4 | arrestin domain containing 4 | n/a | n/a | cell membrane, cytoplasmic vesicle, endosome, plasma membrane | -0.826 | -1.025 |
| BMP6 | bone morphogenetic protein 6 | cytokine, developmental protein, growth factor | chondrogenesis, differentiation, osteogenesis | extracellular region | 0.825 | -1.022 |
| PXMP2 | peroxisomal membrane protein 2 | n/a | n/a | plasma membrane, peroxisome | -0.822 | -1.003 |
| PDK2 | pyruvate dehydrogenase kinase 2 | kinase, transferase | carbohydrate metabolism, glucose metabolism | mitochondrion | 0.821 | -1.015 |
| PDCL2 | phosducin like 2 | n/a | n/a | n/a | 0.819 | -1.015 |
| LDHB | lactate dehydrogenase B | oxidoreductase | n/a | cytoplasm | 0.818 | 1.058 |
| CATSPER4 | cation channel sperm associated 4 | calcium channel, developmental protein, ion channel, voltage-gated channel | calcium transport, differentiation, ion transport, spermatogenesis, transport | plasma membrane | 0.817 | 1.021 |
| RPL15 | ribosomal protein L15 | ribonucleoprotein, ribosomal protein | n/a | plasma membrane | 0.814 | -1.065 |
| CLCC1 | chloride channel CLIC like 1 | chloride channel, ion channel | ion transport, transport | endoplasmic reticulum, golgi apparatus, membrane, nucleus | -0.813 | -1.020 |
| DOC2GP | double C2 domain gamma, pseudogene | n/a | n/a | n/a | 0.811 | -1.011 |
| NKG7 | natural killer cell granule protein 7 | n/a | n/a | plasma membrane | 0.808 | -1.019 |
| ACSS2 | acyl-CoA synthetase short chain family member 2 | ligase | n/a | cytosol | -0.806 | -1.008 |
| CCDC144A | coiled-coil domain containing 144A | n/a | n/a | n/a | 0.804 | -1.013 |
| WDCP | WD repeat and coiled coil containing | n/a | n/a | n/a | -0.804 | -1.034 |

Results of top (p<0.001) Spearman correlation analyses between differentially expressed skeletal muscle genes with improvements in rheumatoid arthritis (RA) (n=12 participants) disease activity score in 28 joints (ΔDAS-28) following high-intensity interval training (HIIT). Fold changes (Δ) of top correlated genes following HIIT are also shown, where positive Δ fold change refers to an increase in expression following HIIT and a negative Δ fold change refers to a decrease in expression following HIIT. Gene molecular process, biological process, and cellular component classifications were identified using Uniprot (www.uniprot.org) keywords. n/a not available

***p<0.05**

Supplementary Table 4. High-intensity interval training rheumatoid arthritis cohort #2: Skeletal muscle canonical pathways associated with improvements in disease activity

| **Ingenuity Canonical Pathways** | **– log**  **(p-value)** | **Ratio (genes/total genes)** | **Positively associated genes** | **Negatively associated genes** |
| --- | --- | --- | --- | --- |
| Proline Biosynthesis II | 3.84 | 4/5 | OAT,PYCR1,PYCR2,PYCR3 |  |
| Arginine Degradation VI | 3.38 | 4/6 | OAT,PYCR1,PYCR2,PYCR3 |  |
| Glycine Cleavage Complex | 3.38 | 4/6 | AMT,GCSH,GLDC,TBXT |  |
| Purine Biosynthesis II | 3.14 | 5/11 | ADSS1,IMPDH1,PAICS | ADSS2,IMPDH2 |
| Proline Biosynthesis I | 2.8 | 3/4 | PYCR1,PYCR2,PYCR3 |  |
| nNOS Signaling in Skeletal Muscle Cells | 2.63 | 9/40 | CACNA1A,CACNA1C,CACNA1I,CACNA2D4, CACNB4,CACNG8,CHRNA1,RYR1 | CACNB1 |
| Calcium Signaling | 2.48 | 26/127 | ATP2B2,ATP2B3,CACNA1A,CACNA1C,CACNA1I, CACNA2D4,CACNB4,CACNG8,CAMK1D,CHRNA1,CHRNA9,HDAC7, MYH6, PRKAG2,RYR1,TRPV6 | ACTC1,CACNB1,MYL1,MYL6,NFATC1,PPP3R1, SLC8A3,TNNC1,TNNT3,TPM3 |
| FcγRIIB Signaling in B Lymphocytes | 2.46 | 13/75 | BTK,CACNA1A,CACNA1C,CACNA1I,CACNA2D4,CACNB4,CACNG8,CD79A | CACNB1, MAPK9,MRAS,RASD1,RRAS |
| Sertoli Cell-Sertoli Cell Junction Signaling | 2.36 | 24/181 | CLDN14,CLDN18,CLDN19,ITGA3,MAP2K3,MYO7A,PRKAG2,PRKG2,SYMPK,TJAP1,TUBB8 | ACTC1,LK,MAP3K6,MAPK9,MRAS,NECTIN1, RASD1,RRAS,SORBS1,SPTBN1,TUBA1A,TUBB2A, TUBB2B |
| L-DOPA Degradation | 2.25 | 2/2 | COMT,LRTOMT |  |
| Noradrenaline and Adrenaline Degradation | 2.24 | 7/30 | ADH1C,AKR1A1,ALDH3A1,ALDH9A1,COMT, LRTOMT | ALDH7A1 |
| Netrin Signaling | 2.08 | 11/65 | CACNA1A,CACNA1C,CACNA1I,CACNA2D4, CACNB4,CACNG8, PRKAG2,RYR1 | CACNB1,NFATC1,PPP3R1 |
| VDR/RXR Activation | 1.94 | 12/77 | HOXA10,HSD17B2,IGFBP3,IGFBP5,KLK6,NCOR2, RXRB,SEMA3B,SULT2A1,WT1 | CASR,TRPV6 |
| Ethanol Degradation II | 1.88 | 6/27 | ADH1C,AKR1A1,ALDH3A1,ALDH9A1 | ACSS2,ALDH7A1 |
| Estrogen Receptor Signaling | 1.84 | 35/319 | AGT,ATP5F1A,ATP5PB,BCL2,CACNA1A,CACNA1C,CTBP1,EGFR,ESR1,GNAO1,HIF1A,MED10,MED14, MED16,MED4, MED6, NCOR2,PLCB1,PRKAG2, TFAM,TYK2,VEGFC | CTBP2,EGF,FOXO6,MMP19,MMP20,MMP21,MRAS,MYL1,MYL6,PLCB2, POLR2B,RASD1,RRAS |
| HIF1α Signaling | 1.84 | 24/200 | APEX1,ARAF,BMP6,CAMK1D,HIF1A,HSPA4, HSPA6,LDHB,MAP2K3,MKNK2,MMP19,MMP20, SLC2A14,SLC2A3,VEGFC | CCNG2,EGF,MDM2,MMP21,MRAS,PPP3R1,RAN,RASD1,RRAS |
| D-glucuronate Degradation I | 1.8 | 2/3 | AKR1A1,DCXR |  |
| Epithelial Adherens Junction Signaling | 1.78 | 19/150 | ACVR2B,EGFR,MYH6,MYO7A,TUBB8 | ACTC1,CRK,EGF,FGF1,MRAS,MYL1,MYL6, NECTIN1,RASD1,RRAS,SORBS1,TUBA1A,TUBB2A,TUBB2B |
| Methylglyoxal Degradation III | 1.77 | 4/14 | AKR1A1,DHRS11 | AKR1C1/AKR1C2,AKR1C3 |
| Estrogen-Dependent Breast Cancer Signaling | 1.72 | 11/73 | DHRS11,EGFR,ESR1,HSD17B11,HSD17B12, HSD17B2 | AKR1C4,MRAS,RASD1,RRAS,TERT |
| G Beta Gamma Signaling | 1.71 | 16/122 | BTK,CACNA1A,CACNA1C,CACNA1I,CACNA2D4, CACNB1,CACNG8,EGFR, GNAO1,GNB1L,PRKAG2 | CACNB4,GNB4,MRAS,RASD1,RRAS |
| Tubby G Protein Signaling | 1.66 | 6/30 | GNB1L,PLCB1,TUB | GNB4,MRAS,PLCB2 |
| Oxidative Ethanol Degradation | 1.66 | 4/15 | ALDH3A1,ALDH9A1 | ACSS2,ALDH7A1 |
| Androgen Signaling | 1.63 | 17/135 | CACNA1A,CACNA1C,CACNA1I, CACNA2D4,CACNB4,CACNG8,DNAJB1, GNAO1,GNB1L,GTF2H5,HSPA4,PRKAG2,SMAD3 | CACNB1,GNB4,MRAS,POLR2B |
| Estrogen Biosynthesis | 1.62 | 7/39 | CYP1B1,DHRS11,HSD17B11,HSD17B12, HSD17B2 | AKR1C3,AKR1C4 |
| Dopamine Degradation | 1.6 | 5/23 | ALDH3A1,ALDH9A1,COMT,LRTOMT | ALDH7A1 |
| Vitamin-C Transport | 1.6 | 5/23 | NXN,SLC2A3,TXNRD3 | AKR1C4,LRRC8A |
| Bladder Cancer Signaling | 1.58 | 13/96 | EGFR,FGF11,MMP19,MMP20,SUV39H1,VEGFC | EGF,FGF1,MDM2,MMP21,MRAS,RASD1, RRAS |
| 2-KG Dehydrogenase Complex | 1.52 | 2/4 | DLST | OGDH |
| Branched-chain α-keto acid Dehydrogenase Complex**^*#^** | 1.52 | 2/4 | BCKDHB,DBT |  |
| Synaptic Long Term Depression | 1.51 | 21/182 | CACNA1A,CACNA1C,CACNA1I,CACNA2D4, CACNB4,CACNG8,CRH,GNAO1,GRM4,PLCB1, PPP2CA,PPP2R3A,PRKG2,RYR1 | CACNB1,GRM3,MRAS,PLAAT4,PLCB2, RASD1,RRAS |
| Neuroprotective Role of THOP1 in Alzheimer's Disease | 1.49 | 14/109 | AGT,APP,GNRH2,GZMA,KLK12,KLK6,PRKAG2, PRSS3,SERPINA3 | FAP,HLA-B,PRSS23,ST14,YWHAE |
| Purine Degradation II | 1.39 | 4/18 | GDA,IMPDH1 | IMPDH2,PNP |
| Glycogen Degradation II | 1.36 | 3/11 | AGL,PGM5 | TYMP |
| GABA Receptor Signaling | 1.33 | 12/94 | ALDH9A1,AP1B1,CACNA1A,CACNA1C,CACNA1I, CACNA2D4,CACNB4,CACNG8, UBQLN1 | CACNB1,KCNN1,MRAS, |
| Lysine Degradation II | 1.32 | 2/5 | AASS | ALDH7A1 |
| Tryptophan Degradation X | 1.31 | 4/19 | AKR1A1,ALDH3A1,ALDH9A1 | ALDH7A1 |
| Ethanol Degradation IV | 1.31 | 4/19 | ALDH3A1,ALDH9A1 | ACSS2, ALDH7A1 |
| 14-3-3-mediated Signaling | 1.31 | 15/126 | PDCD6IP,PLCB1,STRADA,TUBB8,YWHAB | MAPK9,MRAS,PLCB2,RASD1,RRAS,SNCA, TUBA1A,TUBB2A,TUBB2B,YWHAE |

Results of Ingenuity Canonical Pathways analyses, with pathways reaching significance at p>0.05 included. Ratio refers to the number of differentially expressed skeletal muscle genes significantly associated (Spearman correlations, p<0.05) with improvements in rheumatoid arthritis (RA) (RA) (n=12 participants) disease activity score in 28 joints (DAS-28) following high-intensity interval training (HIIT) compared to the total number genes in that pathway.

**^*#^**Pathway significantly associated in both the cross-sectional RA cohort #1 and changes with high-intensity interval training (HIIT) RA cohort #2 analyses
